# Horizontal lang two-pencil test as a screening test for stereopsis and binocularity

**DOI:** 10.4103/0301-4738.64125

**Published:** 2010

**Authors:** Monisha E Nongpiur, Pradeep Sharma

**Affiliations:** Rajendra Prasad Centre for Ophthalmic Sciences, All India Institute of Medical Sciences (AIIMS), New Delhi, India

**Keywords:** Binocularity, Lang two-pencil test, Randot, stereopsis, TNO test

## Abstract

**Purpose:**

To assess the horizontal Lang two-pencil test as a bedside test to detect gross stereopsis.

**Materials and Methods:**

Eighty-four strabismic subjects divided into two groups based on the amount of deviation, and 40 normal subjects were studied. Sensory status examination including binocularity and stereopsis were evaluated with Bagolini, Titmus test and the Netherlands organisation for applied scientific research (TNO), Randot, synoptophore and horizontal Lang two-pencil test.

**Results:**

The subjects in the group with smaller deviation showed better performance on all the four stereo tests and over 90% demonstrated presence of fusion. When compared to TNO and Randot for determining presence of stereopsis, the horizontal Lang two-pencil test demonstrated sensitivity of 100% and 83.9%, specificity of 77.8% and 73.7%, and negative predictive value of 100% and 100% respectively. It also showed 100% specificity as a test for binocularity when compared with the Bagolini striated glass test.

**Conclusion:**

Horizontal Lang two-pencil test, an easily performed test with a high sensitivity and negative predictive value can be used as a screening test to detect gross stereopsis and binocularity.

Stereopsis is defined as the highest level of binocular interaction and requires a good visual acuity in each eye, simultaneous perception, and fusion. When strabismus is acquired later in life, the loss of stereopsis is felt acutely and may present a real handicap.[1] It is useful to determine whether a patient with strabismus has stereopsis or the potential for such.[2‐5]

Amongst the stereo tests,[6‐11] the Lang two-pencil test, which is a simple, easily performed test to assess gross stereopsis, has not been extensively used as an adjunct to the orthoptic/ ophthalmological evaluation of strabismic patients or as a screening test for stereopsis. The advantage of the Lang two-pencil test is that it does not require any special instruments/ equipments like goggles and test plates and it can be easily performed in younger children. Pugesgaard *et al*.[11] in a comparative study of a Rodenstock sight screener and a clinical eye examination had incorporated an orthoptic examination that included the Lang two-pencil test, Titmus test and the Netherlands organisation for applied scientific research (TNO) stereotest and the Bagolini striated glass test. They found that any of the tests could be used as a supplement to the ophthalmological examination to pick up an orthoptic pathology, which would then require detailed evaluation. We feel there is a possibility of false positive error if the pencils are held vertically because then the task could also be done monocularly, if the subject sees the tip of the examiner's pencil 'end-on'. The purpose of this study was to assess the Lang two-pencil test used horizontally as a test to detect gross stereopsis and to compare it with the TNO and Randot stereo-acuity tests.

## Materials and Methods

This was a cross-sectional study evaluating the horizontal Lang two-pencil test in acquired strabismus and normal subjects after approval from the Institutional review board of our Institute. The subjects were enrolled from the squint clinic and outpatient department of our Institute. The inclusion criteria were subjects aged six years and above, with an onset of deviation after the first year of life, best corrected visual acuity (BCVA) of 20/60 or better in each eye at the time of examination, and neurologically and developmentally normal. Any subject with vertical strabismus more than 5 prism diopters (PD), or with intermittent deviation or any heterophoria, or not willing to participate in the evaluation was excluded. Patients with intermittent strabismus were excluded because it is believed that they may have some fusional component. Eighty-four subjects met the inclusion criteria. The subjects were divided into two groups based on the amount of deviation; Group 1 with an angle of deviation less than or equal to 8 PD, and Group 2 with deviation greater than or equal to 10 PD. There were 40 normal subjects. The criteria for normal subjects were a BCVA of 20/20 with no significant anisometropia, and who were orthophoric. Informed consent was obtained from the subjects or parents.

A history provided by the parents or guardians detailing the age of the patient, age of onset of deviation, previous therapy including optical, surgical and amblyopia treatment, age at alignment, and whether optically or surgically realigned, was noted. Clinical evaluation included visual acuity testing, refraction, ocular movement assessment, cover test, and prism bar cover test (PBCT) to determine the amount of deviation. Sensory status examination for binocularity and fusion were performed using the Bagolini striated glass test (a routinely used test), the three circles test on TNO and the R and L test in the Randot booklet. Stereopsis was assessed by the synoptophore, horizontal Lang two-pencil test, TNO and Randot test booklets. TNO or Randot are routinely used stereo tests to determine level of stereo-acuity.

Visual acuity was assessed using the Snellen's visual acuity chart. Bagolini glass test was used to assess binocularity and fusion consists of lenses with striations which are at right angles to each other. It was performed by keeping a pair of the striated glasses in front of each eye, and the subject was asked to fixate on a punctate light source. The possible responses were a) symmetrical cross response b) asymmetrical cross response c), single line response, and d) cross response with a central gap in one line. Responses 'a' and 'b' indicate binocularity while response 'c' indicates suppression. The central suppression response 'd' indicates either a fixation point scotoma (with manifest deviation and anomalous retinal correspondence) or foveal scotoma (with orthophoria and normal retinal correspondence). The three circles test on TNO and the R and L test in the Randot booklet was explained together with the respective tests.

**Figure 1:**
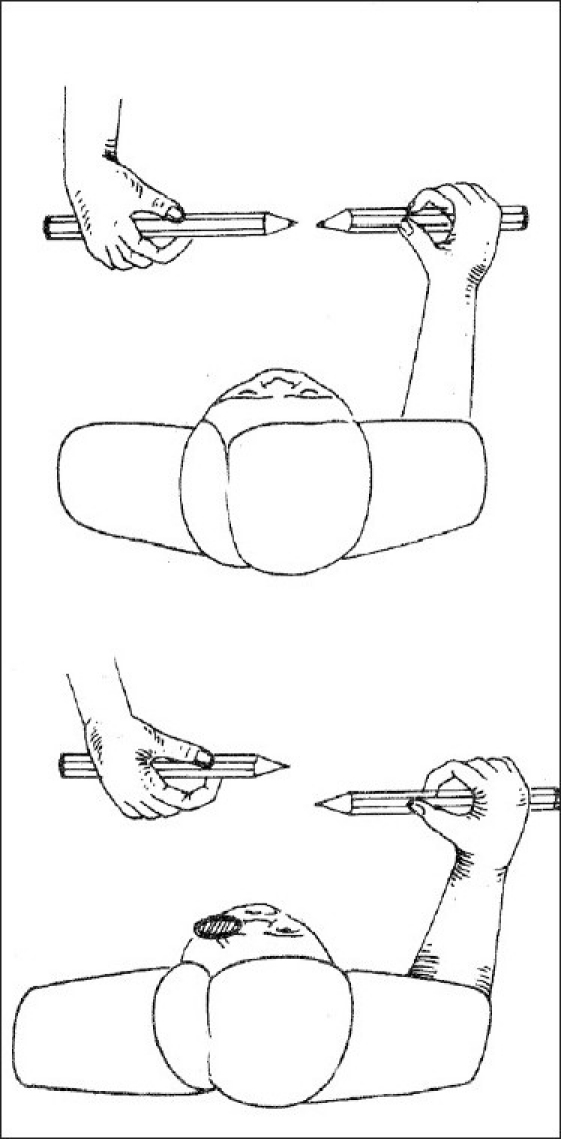
Illustration of the horizontal Lang two-pencil test

Gross stereopsis was determined by synoptophore using stereopsis slides, and the horizontal Lang two-pencil test. The synoptophore consists of two cylindrical tubes placed horizontally and contains a +6.0 or a +6.5D lens in each eyepiece which sets the target distance to about 6 meters. Stereopsis was detected as the ability to obtain the impression of depth by the superimposition of two pictures of the same object taken at a slightly different angle. The different stereopsis slides used were that of a bucket; a clown with several objects about him. In the horizontal Lang two-pencil test, an examiner held a pencil horizontally at the eye level in front of the subject in the middle, who, with another pencil was asked to touch the tip of the examiner's pencil with the tip of his/ her pencil [Fig. 1]. The test was considered positive when the subject was able to touch the tip of the examiner's pencil. The test was conducted by a single examiner. Care was taken to prevent the patient from seeing the tip of the examiner's pencil 'end on', because then the test could be performed monocularly, which can easily be possible if the pencils are kept vertical below the eye level. The stereo-acuity detected by the Lang two-pencil test is of the order of 3000-5000 sec of arc.[1]

The level of stereo-acuity was evaluated using TNO and Randot stereo tests, which are based on the Random dot stereograms. They contain no information about the shape or nature of the object hidden in the visual noise of random dots. Only when the images from the right and left eye are combined at the neural level and the object is seen in depth does recognition take place.

The TNO stereo test was performed by placing the plates at a test distance of 40 cm squarely in front of the patient, wearing spectacle correction if using one. The test consists of seven plates, the first three of which were used for screening to detect the presence of gross stereopsis. The fourth plate was a suppression test. The last three plates on the other hand were used to quantitatively assess stereo-acuity in the range of 15 to 480 sec of arc. Each plate consisted of four squares, within which was a circle with a 'pie'/ piece missing. The subjects were asked to point to the missing piece. This test required the use of red-green glasses. The normal value for stereo-acuity was considered ≤60 sec of arc.

The Randot stereo test was administered by holding the test booklet upright before the subject, about 40 cm away. The test required the use of polarizing glasses to be worn over prescription glasses, if using any. The Randot stereo test consisted of three parts–geometrical shapes, animals, and circles with a disparity range of 500-250, 400-100, and 400-20 sec of arc respectively. The booklet also contained a test for suppression in the form of an R, L and a cross.

Any level of measurable stereo-acuity on the Randot (up to 500 sec of arc) or TNO (up to 480 sec of arc) was considered as *presence of stereopsis*. False positive is defined as the absence of measurable stereo-acuity on the TNO or Randot, but presence of gross stereopsis on the synoptophore or horizontal Lang two-pencil test.

### Statistical analysis

Mean age and gender ratio between the groups was compared using student t-test and chi square test respectively. *P* value of < 0.05 was considered as statistically significant.

## Results

Of the 84 strabismic subjects there were 56 males and 28 females aged between 6 and 19 years with a mean age of 9.13 ± 3.38 years. Of the 40 normal, there were 23 males and 17 females ranging in age from 6 to 17 years with a mean age of 9.7 ± 2.98 years. There was no statistical difference between the strabismic and normal subjects in mean age and gender ratios (*P*=0.3 for both). The type of strabismus and amount of deviation is given in detail in Table 1.

**Table 1:** Type of strabismus and amount of deviation

	2 PD	4 PD	6-8 PD	10 PD	12-24 PD	≥25 PD
Accommodative ET	10	6	5	0	0	0
Partially accommod. ET	0	0	0	6	3	2
Non-accommodative ET	0	0	0	0	1	7
Treated anisometropic amblyopia with ET	0	2	1	2	0	0
Residual ET	3	7	1	4	3	1
Consecutive ET	0	0	0	0	1	0
Treated anisometropic amblyopia with XT	3	2	1	0	0	0
Consecutive XT	2	0	1	0	1	0
Unilateral /alternating divergent squint	0	0	0	0	0	9

ET: Esotropia XT: Exotropia PD: Prism diopter

Performance of the subjects on the Bagilini striated glass test and the four stereo tests is summarized in Table 2. Over 90% of subjects in the group with a smaller angle of deviation, and all normal subjects showed presence of fusion, whereas only 30% of subjects with larger deviation had fusion. Since Bagolini detects binocularity and not stereopsis, it was therefore not included in the tests for stereopsis. As expected, subjects with lesser deviation demonstrated presence of stereopsis as determined by the various stereo tests.

**Table 2:** Evaluation of sensory status in strabismic and normal subjects

Test	Group 1 (n=44)	Group 2 (n=40)	Normal (n=40)
Binocularity: Bagolini striated glass test			
Fusion	40 (91)	12 (30)	40 (100)
Suppression	4 (9)	18 (45)	0 (0)
Variable response	0 (0)	10 (25)	0 (0)
Stereopsis			
Horizontal Lang twopencil test	30 (67)	8 (20)	40 (100)
Synoptophore	25 (57)	2 (5)	40 (100)
TNO	25 (57)	1 (2.5)	40 (100)
Randot	25 (57)	1 (2.5)	40 (100)

Figures in parentheses are in percentage

The mean, median and range of the stereo-acuity level on the TNO and Randot in Group 1 were 161.5 ± 117, 120, and 60– 480 sec of arc; and 107.88 ± 114, 70, and 25 – 400 sec of arc respectively; while in the normal subjects it was 67.5 ± 26, 60, and 30 – 120 sec of arc; and 42.5 ± 19, 40, and 25–70 sec of arc respectively. The lone patient in Group 2 who had measurable stereo-acuity had a deviation of 10 PD; the age of onset of deviation was four years, and surgery was performed at the age of 4.67 years. The duration of deviation prior to surgery was therefore 0.67 years (eight months). The patient was diagnosed to have partially accommodative esotropia, with a deviation of 25 PD prior to surgery. The patient's hyperopic correction was 4.37 diopters spherical equivalent.

A subgroup analysis was done for the subjects in Group 1 between those with and without stereopsis. There were 26 subjects with stereopsis (25 had measurable stereo-acuity levels and one had only gross stereopsis) and 18 without stereopsis. Of the 26 with stereopsis, two were missed on the synoptophore. One of these patients had stereo-acuity of 480 arc seconds on the TNO and the other had stereo-acuity of 400 arc seconds on the Randot. However, in those without stereopsis, all but one patient (false positive) showed absence of stereopsis on the synoptophore. Evaluation using the horizontal Lang two-pencil test showed that all the patients with stereopsis were picked up correctly and there were four false positive results. There were no false negative results. Examination using the Bagolini striated glass test for binocularity showed that all the patients with stereopsis had binocular single vision. However, 14 of the 18 patients with no measurable stereo-acuity also showed presence of binocular single vision (data not shown). The study was to compare any level of measurable stereo-acuity on the two stereo tests, namely TNO and Randot, with the gross stereopsis detected by the horizontal Lang two-pencil test. Therefore, comparison of patients with random dot versus local stereopsis was not performed.

Table 3 shows the predictivity of the synoptophore and horizontal Lang two-pencil test in comparison to TNO and Randot as a test for stereopsis. In Table 4, binocularity was compared between the three circles' test on TNO, R and L test on Randot and Lang two-pencil test taking Bagolini striated glass test as the gold standard for binocularity. As can be seen in the Table, Lang pencil test being a test to detect gross stereopsis, shows 100% specificity for binocularity.

**Table 3:** Predictivity for stereopsis of the horizontal Lang two-pencil test taking TNO and Randot separately as the gold standard

	Sensitivity %		Specificity %		PPV %		NPV %	
	TNO	Randot	TNO	Randot	TNO	Randot	TNO	Randot
Lang two-pencil	100	83.9	77.8	73.7	87.1	83.9	100	100

**Table 4:** Predictivity for binocularity of Lang two-pencil, TNO and Randot taking Bagolini as the gold standard

	Sensitivity	Specificity	PPV	NPV
Lang two-pencil	75.6	100	100	28.6
TNO (three-circle test)	97.6	75	97.6	75
Randot (R,L test)	97.6	75	97.6	75

Values are given in percentage

## Discussion

In the current study, we describe the horizontal Lang pencil test as a parameter to evaluate gross stereopsis and compare it with the currently and commonly used stereo tests. We found that the easily performed horizontal Lang two-pencil test detected all subjects who had stereopsis. In addition, with no false negative responses and a 100% sensitivity and negative predictive value (NPV), this test may be used as a potential screening test for detecting gross stereopsis.

Stereo-acuity has been measured by various tests and there are conflicting reports on their validity in the evaluation of strabismic and amblyopic patients. We found that the median in the normal subjects was 60 sec of arc on TNO and 40 sec of arc on Randot while in the strabismic subjects, the median was 120 and 70 sec of arc respectively. Similar observations were made by other authors.[7,12] Garnham and colleague[12] in their evaluation of the effect of age on stereo-acuity found that TNO was significantly different from the other stereo tests. They noted the following median values: TNO, 60 sec of arc; Titmus, 40 sec of arc; Frisby near, 30 sec of arc; Frisby-Davis distance, 15 sec of arc. A similar trend was noted by Marsh *et al*.[7] who had evaluated normal and strabismic-anisometropic groups of children using four stereo tests: RDE, Randot, Titmus and TNO. They also found a wide variation in the test means, and had suggested the following explanation. The intrinsic disparity range was different for different tests, where the test with the most figures in the lower disparity range, should give the lowest mean.

The horizontal Lang two-pencil test, a subjective test which could be easily performed, was able to detect all the patients with stereopsis. It was interesting to note that it also detected stereopsis in four of the 18 patients who otherwise had no measurable stereopsis on TNO and Randot. These four subjects with false positive responses did show binocular single vision on the Bagolini, TNO and Randot, hence they were likely to have had stereopsis of the order of 3000 arc sec detected by the Lang two-pencil test alone.[1] We found no false negative responses with the test.

The Lang two-pencil test has not been extensively studied as an adjunct to the orthoptic/ ophthalmological evaluation of strabismic patients, or as a screening test for stereopsis. Pugesgaard *et al*.[11] noted that Lang two-pencil test or TNO stereo test or Bagolini striated glass test could be used as a supplement to the ophthalmological examination to pick up orthoptic pathology, which would then need detailed evaluation. With a 100% sensitivity and NPV, its potential as a screening test is immense. The Lang two-pencil test by Lang, was originally described to be performed by approaching the rod vertically so as to mimic the daily tasks which requires stereopsis, such as pouring liquid into a tumbler, or hitting a nail. But these tasks could also be done monocularly, if the subject sees the top of the tumbler or the tip of the examiner's pencil 'end-on' in the vertically held pencil test. This can be more easily avoided if the test is performed by approaching the rod/ examiner's pencil horizontally at the eye level of the subject. We believe that the horizontal Lang two-pencil test gives a more accurate assessment for the detection of gross stereopsis. However, this point has not been proven and a comparative evaluation between the vertical and horizontal pencil test has not been done. Evaluation using the synoptophore is more complicated, and we found it to be not as sensitive as the horizontal Lang two-pencil test. A possible reason could be the younger age group of our subjects who may have found the test trying and not easy to perform.

There were some limitations to our study. First, we have not attempted to compare the horizontal Lang two-pencil test with the popularly performed vertical test, hence our assumption that the horizontal test contains less monocular cues could not be proven. Second, the pencil test is a qualitative test, it should be used purely for screening especially in situations where quantitative tests like the TNO, Randot and Frisby are not readily available.

In conclusion, the horizontal Lang two-pencil test, an easily performed test even in children, with a high sensitivity and negative predictive value can be used as a potential bedside test to detect stereopsis and binocularity.
